# Could a simple manual technique performed by a midwife reduce the incidence of episiotomy and perineal lacerations? A non-randomized pilot study

**DOI:** 10.18332/ejm/191749

**Published:** 2024-09-05

**Authors:** Kathryn E. Taylor, Virginia Stulz

**Affiliations:** 1 Nepean Hospital, Kingswood, Australia; 2 Faculty of Health, University of Canberra, Bruce, Australia

**Keywords:** perineum, myofascial release, vaginal birth, labor, episiotomy

## Abstract

**INTRODUCTION:**

Women experience medical interventions, episiotomy, and perineal lacerations during childbirth, impacting their physical, psychological, and sexual well-being. This study compares the perineal status of prospective women who had the midwifery intervention of perineal myofascial release during childbirth, to a matched retrospective control sample of women who received standard care during childbirth.

**METHODS:**

A non-randomized pilot study with prospective data collected for 50 women after informed verbal consent was obtained to having the midwifery intervention of perineal myofascial release during childbirth, and the matched retrospective data for the control group of 49 women were collected from a random sample generated from the medical records. Quantitative analyses included descriptive statistics, independent t-tests, regression, and chi-squared analyses. Retrospective trial registration was granted with The Australian New Zealand Clinical Trials Registry ANZTR.

**RESULTS:**

Women were six times (OR=0.15; 95% CI: 0.0–0.37) less likely to have a non-intact perineum and twice (OR=0.44; 95% CI: 0.35–0.56) less likely to have an episiotomy if they were in the intervention group. Chi-squared analysis found no statistically significant differences between groups for normal vaginal birth and instrumental births, excluding cesareans and waterbirth [χ^2^(1)= -0.37, p=0.542].

**CONCLUSIONS:**

This study found perineal myofascial release benefits women by reducing perineal trauma and episiotomy. However, there were no significant differences in the duration of the active pushing stage of labor or mode of birth. This study has shown some promise in obtaining data for a larger, definitive, randomized controlled trial.

**CLINICAL TRIAL REGISTRATION:**

The study was registered on the Australian New Zealand Clinical Trials Registry ANZTR.

**IDENTIFIER:**

ID ACTRN12623000807651

## INTRODUCTION

Normal vaginal birth and protection of the perineum is a priority for midwives to ensure positive birth experiences and outcomes for women. In Australia in 2019, one in five women birthed with an intact perineum, about a quarter (23%) experienced a first-degree laceration, while nearly one-third (31%) experienced a second-degree laceration, and a small (<3%) number of women experienced a third- or fourth-degree laceration^1^. Alarmingly, one in four women sustained an episiotomy (23.2%) during birth, 12.6% had an instrumental birth, and of these, 82% were nulliparous mothers sustaining an episiotomy^1^. The World Health Organization (WHO) recommends episiotomies should not be routinely used and does not stipulate a reasonable episiotomy rate^2^.

Perineal trauma during birth can significantly impact a woman’s quality of life due to perineal pain, infection, or dehiscence^3^ and dyspareunia^4,5^. Severe trauma may result in urinary or fecal incontinence with pelvic organ prolapse^6-8^ or a puborectalis avulsion^9^. Alperin et al.^10^ found episiotomy increases a woman’s risk of severe perineal trauma in future births. The psychological birth trauma women experience due to perineal lacerations or episiotomy can lead to post-natal depression, post-traumatic stress disorder (PTSD)^4,11,12^ and sexual dysfunction^5^.

This non-randomized pilot study aimed to compare the perineal status of women (n=50) with perineal myofascial release during childbirth to a matched control group of women (n=49) who received standard care during childbirth. Our research question was: ‘Were there any differences between the length of the active pushing stage of labor, mode of birth, perineal status, and genital tract trauma for women having perineal myofascial release during the active pushing stage of labor (intervention group) in comparison to women who did not have perineal myofascial release (control group)?’.

## METHODS

### Study design and setting

This is a non-randomized pilot study. Prospective and retrospective data from October 2020 to August 2022 were collected, which included 50 women who received perineal myofascial release during the active pushing stage of labor (intervention group) and 49 women who received standard care during the active second stage of labor and did not receive perineal myofascial release (control group). The intervention can only be used with ‘the fetal head on view’ (crowning) as the technique would be inappropriate prior to the fetal head being on view (crowning). The control group (n=49) included a matched random sample of women who gave birth between October 2020 and August 2022, which was generated from medical records. A waiver of consent was obtained for the comparison group data. Both the intervention and the control groups had warm compresses applied to the perineum during the active pushing stage of labor. This study was conducted in the birth unit of a tertiary public hospital in metropolitan New South Wales, which has more than 4000 births per year. We matched the intervention and control groups on their age, parity, gestation, and body mass index (BMI, kg/m^2^).

### Intervention

**Figure. 1:**
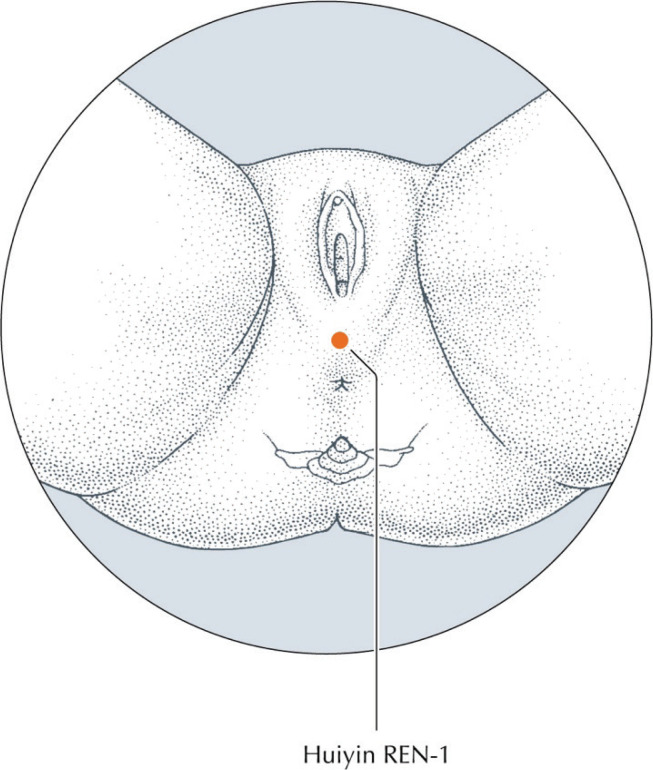
Location of Conception Vessel 1 (CV1) at the center of the perineum (reproduced with permission from Deadman et al.^12^)

Conception Vessel 1 (CV1), translated from the pinyin Ren 1 *huiyin* 會陰, is located at the midpoint of the perineum (Figure 1) at the center of the perineal body between the vagina and the anus^13^. The perineal body is an attachment point or conduit of the pelvic floor’s interlaced and interdependent perineal muscle fibers, tendons, and fascia^14,15^. CV1 is located by the midwife with flat finger palpation^16^ along the length of the perineum until a nodule or ‘knot’ is felt^16-18^, or CV1 is visualized as a ‘dimple’ in the center of the perineum once the fetal head is on view exerting force upon the pelvic floor.

The perineal myofascial release technique involves the midwife applying continual digital pressure to CV1 using the middle or index finger of their dominant hand when the fetal head is visible and low on the perineum. Simultaneously, the midwife places their non-dominant hand on the baby’s head to apply counter-pressure. Perineal myofascial release is a slow and controlled hands-on technique integrated into the current practices outlined in the Perineal Protection Bundle^19,20^. The midwife commences perineal myofascial release when the fetal head is on view, and no further progress of descent or extension is observed due to a perineal tight band and the indication for an episiotomy. The technique was applied for a minimum of one pushing contraction to a maximum of eight pushing contractions.

The intimate location of CV1 prohibits use by acupuncturists, is rarely used in acupuncture clinical practice, and is contraindicated for use during pregnancy^13^. However, midwives are uniquely placed with birthing women, and the novel use of CV1 to facilitate birth and prevent perineal trauma is being developed at an Australian tertiary hospital.

Seven birth unit midwives were trained in the perineal myofascial release technique, with a 30-minute in-service education package followed by the midwife applying the technique as the accoucheur at birth.

### Sampling

The sample size included 99 women in total. Prospective data were collected for 50 women after informed verbal consent was obtained to have the midwifery intervention of perineal myofascial release during childbirth.

*Inclusion criteria*

The inclusion criteria for the intervention group (n=50) required that the mother had a term (>36 weeks gestation) singleton pregnancy who birthed vaginally, after verbal informed consent to receive perineal myofascial release during the active pushing stage of labor by a midwife trained in the technique, had a baby between October 2020 and August 2022, and was aged ≥18 years. One woman with a term known as fetal death *in utero* was included in this study.

*Exclusion criteria*

The exclusion criteria were women who had a waterbirth, a pathological cardiotocograph (CTG)^2^, fetal bradycardia, shoulder dystocia, perineal button-holing, or infectious lesions on the perineum.

### Ethics

The Human Research Ethics Committee approved this study from the 2020/ETH02967 hospital, where the research was conducted, and the University Ethics - RH14240, and informed verbal consent was approved for the study intervention group. The original ethics approval was for antenatal written informed consent. However, due to COVID-19 restrictions in the hospital, we could not consult women antenatally, and a new ethics amendment was obtained for verbal consent when presenting to the birth unit. Therefore, women verbally consented to the technique at the time they presented to the hospital in labor. A waiver of consent was approved for the retrospective quantitative data (control group data).

### Data measurements

Prospective and retrospective quantitative data were collected during this study to show the differences between the application of perineal myofascial release or without perineal myofascial release during the active pushing stage of labor. Perineal status was identified by the accoucheur in consultation with a senior midwife or obstetric registrar. Reviews of e-Maternity and Powerchart, and electronic health records provided information on maternal age, BMI, gestation, parity, length of second stage, mode of birth, and perineal status. Two groups were formed: those with perineal myofascial release (intervention group, n=50) and those without (control group, n=49). The duration of perineal myofascial release applied by the midwife could not be determined due to the lack of information in the medical record.

### Data analysis

Descriptive statistics provided information by way of percentages for parity and perineal status. Independent t-tests were conducted to compare the two samples’ continuous variables (age, body mass index, gestation, active pushing). Means and standard deviations are also presented for the two samples. Chi-squared analyses and odds ratios were conducted to establish women who were more likely to have an intact perineum or episiotomy.

## RESULTS

### Characteristics of the study sample

We have included the characteristics of the sample in Table 1. An independent t-test found no significant differences between groups for age, BMI, gestation, and active pushing stage of labor.

**Table 1:** Demographic and clinical characteristics of women who had midwifery intervention of perineal myofascial release during childbirth (intervention group) and women who did not have (control group), October 2020 to August 2022, Australia (N=99)

*Characteristics*	*Intervention group (N=50)*		*Control group (N=49) Mean ± SD*
	*Mean ± SD*	*p*	
Age (years)	28.1 ± 5.3	0.656	28.5 ± 4.7
BMI	24.9 ± 5.8	0.868	24.7 ± 5.4
Gestation (weeks)	39.1 ± 1.2	0.358	39.3 ± 1.4
Active pushing duration (minutes)	34.7 ± 26.3	0.616	31.7 ± 33.2
Multipara, n (%)	28 (56)		31 (63.26)
Nullipara, n (%)	23 (46)		17 (34.69)

BMI: body mass index (kg/m^2^).

### Mode of birth

Chi-squared analysis found no statistically significant differences between groups for a normal vaginal birth and instrumental births, excluding cesareans and waterbirth [χ^2^(1)= -0.37, p=0.542; OR=2.09; 95% CI: 0.18–23.77].

### Intact perineum and episiotomy

**Figure. 2:**
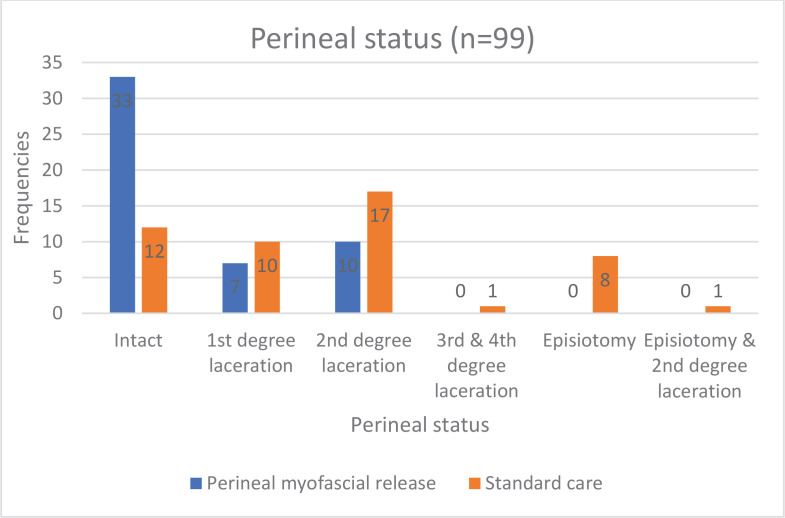
Perineal status for women who had midwifery intervention of perineal myofascial release during childbirth (intervention group) and women who did not have (control group), October 2020 to August 2022, Australia (N=99)

Chi-squared analyses in the intervention group with perineal myofascial release revealed a significant association between having the technique and having an intact perineum [χ^2^(1)=18.37, p<0.001]. Chi-squared analyses in the intervention group also revealed a significant association between having perineal myofascial release and not having an episiotomy [χ^2^(1)=10.10, p<0.001]. Based on the odds ratio, women (n=50) in the intervention group were six times (OR=0.15; 95% CI: 0.06–0.37) less likely to have a non-intact perineum and twice (OR=0.44; 95% CI: 0.35–0.56) less likely to have an episiotomy. Figure 2 shows the frequencies for perineal status.

## DISCUSSION

This study provides initial evidence of the potential benefits of perineal myofascial release during the active pushing stage of labor for birthing women. Our study found women who have perineal myofascial release during the active pushing stage of labor are more likely to have an intact perineum and are less likely to have an episiotomy. Current literature on the prevention of perineal trauma during birth is divided between two areas of discussion, the hands-on birth versus the hands-off birth. A hands-on birth technique defined by Pierce-Williams et al.^21^ describes one hand on the fetal head applying counter pressure to prevent a quick birth. The other hand applies pressure on the mother’s perineum with various manual maneuvers or applications^9,22^. A hands-off or hands-poised birth implies no touching of the fetal head or the mother’s perineum during birth, which is usual practice for a waterbirth or a standing birth^9,21,22^.

The evidence-based hands-on birth technique that midwives currently use in Australia to prevent perineal trauma is the application of a warm compress to the perineum during birth. Three systematic reviews^7,8,23^ found good evidence to support warm compress application to the perineum during the crowning of the fetal head to prevent third- and fourth-degree lacerations. Yet, Aasheim et al.^8^ cautioned a hands-on birth may increase episiotomy. Our study found that women who had perineal myofascial release during birth did not experience an episiotomy or severe perineal trauma, and they were more likely to give birth with an intact perineum. Perineal myofascial release was integrated into the standard care provided during birth by applying a warm compress to the perineum^19,23^. The technique was further developed by alternating a warm compress to provide comfort to the woman during the push and then removing the warm compress to apply for perineal myofascial release after the push to release the transverse perineal muscles, providing more space for the baby to birth without the need for an episiotomy.

Maternal positions are an intrapartum risk factor that can increase the risk of perineal trauma and episiotomy. Studies have identified that maternal positions of lying supine, semi-recumbent, or lithotomy were more likely to require an instrumental birth with an episiotomy^24,25^. In our study, however, midwives could only visualize and access the perineum to correctly apply the perineal myofascial release technique if the woman was in a semi-recumbent, supine, lithotomy, or lateral position. This suggests the hands-on birth technique of perineal myofascial release is most appropriate for women who have reduced mobility due to regional analgesia or who choose a semi-recumbent, supine, or lateral position.

Midwives in our study were unable to access or correctly apply the technique in maternal upright positions, with Lodge and Haith-Cooper^26^ finding the maternal birth positions of kneeling or on all fours as most protective for an intact perineum, therefore perineal myofascial release would not be necessary. Maternal effort may be another risk factor, with exhaustion lengthening the duration of the second stage of labor that correlates with medical intervention and severe perineal trauma^6,27^. Midwives in our study found that perineal myofascial release facilitated progress in the second stage of labor. However, the active pushing stage of labor time we compared between our study’s intervention and control groups showed no significant differences. The study found no difference between the groups in terms of mode of birth.

Two possible mechanisms of action to explain perineal myofascial release are that it may enhance both a physiological and biomechanical response during birth. Firstly, Ferguson’s reflex, a neuroendocrine positive feedback response due to biomechanical pressure exerted onto the perineal soft tissues by the fetal head, signals the posterior pituitary to increase oxytocin to boost expulsive uterine contractions^28^. Perineal myofascial release may be especially beneficial to facilitate birth without an episiotomy when maternal exhaustion^6,24^ or regional analgesia^25^ prolongs the active pushing stage of labor.

The second possible action is that perineal myofascial release has a biomechanical effect on all pelvic floor muscles that insert or are continuous with the perineal body^14,15^, providing extra stretch and contractility, allowing more space for the baby to birth. Myofascial trigger point release with acupuncture has been shown to relax hypertonic pelvic floor muscles^18^. Dietz^9^ identified a more elastic levator ani muscle as being associated with a shorter second stage of labor, and Berghmans^17^ explains that myofascial trigger point release restores the proper length to the pelvic floor muscles. Therefore, perineal myofascial release on CV1 could provide this extra elasticity by improving the contractility of the pelvic floor to facilitate normal vaginal birth. Contraindications identified by the midwives for perineal myofascial release included abnormal fetal welfare^20^ that required immediate escalation of care and an instrumental birth with a selective mediolateral episiotomy^19,27,29^. Other contraindications included a handsoff birth such as a waterbirth, standing birth, or maternal request for no touching.

Evidence exists for midwives to apply acupressure during labor for pain management, reducing the need for pharmaceutical analgesia, increasing women’s satisfaction with their birth experience^30^, and shortening the first stage of labor^31^. The acupuncture point CV1 stimulated with perineal myofascial release by the midwife during the active pushing stage of labor may have the potential to benefit birthing women by reducing the episiotomy rate and increasing an intact perineum. While previous research has focused on warm compresses to prevent third- and fourth-degree lacerations, these results demonstrate the potential to manipulate the physiology and biomechanics of birth to reduce the incidence of episiotomy and increase an intact perineum.

### Limitations

The nature of this study could not randomize the intervention group and introduces more threats regarding internal validity. However, one way we dealt with these problems was by matching. In this way, we systematically identified women in the intervention or comparison groups based on whether or not they had perineal myofascial release during birth. We sought to ensure they did not differ on other key variables, such as age, BMI, parity, and gestation^21^. Another limitation is that we did not adjust for other known factors for perineal lacerations, such as birth weight and ethnicity^21^. One limitation of the study was that it was conducted in one hospital in Australia, and the results may not be generalizable to other settings. The women were not followed up to ask for their experience and if it was tolerable for them to have the technique during their birth due to COVID-19 restrictions in the hospital. Further research is needed to ask the women about their experience of perineal myofascial release. Also, neonatal outcomes were not included in this study and may be necessary for the development of guidelines for the safe practice of perineal myofascial release. Further definitive research, should be undertaken with a randomized controlled trial that should include more refined analyses such as logistic regressions that could adjust for other known factors of perineal lacerations.

## CONCLUSIONS

This study found perineal myofascial release benefits women by reducing perineal trauma and episiotomy. However, there were no significant differences in the duration of the active pushing stage of labor or mode of birth. This study has shown some promise in obtaining data for a larger, definitive, randomized controlled trial.

## Data Availability

The data supporting this research are available from the authors on reasonable request.
